# Job Satisfaction, Self‐Rated Work Performance, and Precuneus Gray Matter Volume: An Exploratory Neuroimaging Study

**DOI:** 10.1002/brb3.71597

**Published:** 2026-07-14

**Authors:** Keisuke Kokubun, Kiyotaka Nemoto, Yoshinori Yamakawa

**Affiliations:** ^1^ Graduate School of Management Kyoto University Kyoto Japan; ^2^ Department of Medical Informatics and Management and Psychiatry, Institute of Medicine University of Tsukuba Tsukuba Japan; ^3^ Institute of Innovative Research Institute of Science Tokyo Meguro Japan; ^4^ ImPACT Program of Council for Science Technology and Innovation (Cabinet Office, Government of Japan) Chiyoda Japan; ^5^ Office for Academic and Industrial Innovation Kobe University Kobe Japan; ^6^ Brain Impact Kyoto Japan

**Keywords:** gray matter volume, job satisfaction, precuneus, work performance

## Abstract

**Introduction:**

In today's world of intensifying global competition, it is becoming increasingly important to enhance employees' job performance. However, the relationship between brain structure and job performance is unclear.

**Methods:**

Therefore, we hypothesized and tested whether the gray matter volume in the precuneus, which is involved in metacognition and the default mode network, statistically accounts for part of the association between job satisfaction and job performance through the mediation analysis.

**Results:**

This study showed that the gray matter volume of the precuneus statistically accounts for part of the association between job satisfaction and work performance. Furthermore, exploratory analysis showed that the superior parietal lobule and midcingulate cortex may be related to work performance, and the superior temporal sulcus, rectus, medial orbitofrontal cortex, amygdala, and pallidum may be related to job satisfaction.

**Conclusion:**

These results are the first to show that the gray matter volume of brain regions, including the precuneus, which is responsible for functions such as metacognition, reflection, creativity, and self‐efficacy, is related to job satisfaction and work performance, consistent with the self‐regulated learning strategy theory.

## 1 Introduction

In today's world of intensifying global competition, it is becoming increasingly important to enhance employees' job performance. Therefore, various factors of job performance have been examined and have become the subject of research. A representative theory is Zimmerman's (2000) self‐regulated learning (SRL) strategy theory. SRL includes cognitive, metacognitive, behavioral, motivational, and affective/emotional aspects of learning, and the model consists of three phases: foresight, performance, and self‐reflection. In the foresight stage, individuals analyze the task, set goals, and plan how to reach them, and many motivational beliefs activate the process and influence the activation of learning strategies. In the performance phase, individuals actually perform the task while monitoring their own progress and using several self‐regulation strategies to maintain cognitive engagement and motivation to complete the task. Finally, in the self‐reflection stage, individuals evaluate how they performed the task and make attributions about success or failure. These attributions generate self‐reactions that can have a positive or negative impact on how individuals approach the task in their later performance. Previous studies have shown that SRL can explain variance in sports (Cleary and Zimmerman 2001; Cleary et al. 2006; Kitsantas and Zimmerman 2002) and academic (DiBenedetto and Zimmerman 2010; Wu et al. 2023; Zhao et al. 2025) performance, as well as work performance (Cuyvers et al. 2020; Van Loon 2018).

Performance has also been the subject of brain research. For example, previous studies have found positive correlations between the parahippocampal gyrus and verbal memory, between the medial temporal lobe and putamen and visuospatial memory, and between the middle temporal gyrus and frontal lobe and verbal fluency in patients with Parkinson's disease (PD) (Gerrits et al. 2014). On the other hand, studies in healthy adults have shown that gray matter volume (GMV) correlates with cognitive performance (Haier et al. 2010; Narr et al. 2007). However, the relationship between work performance and brain structure in healthy middle‐aged people has not been addressed so far. In light of SRL strategy theory, unlike brain science studies conducted in laboratories, work performance is thought to improve through continuous learning, including evaluation and reflection on one's own work, so a relationship with brain regions related to metacognitive ability is expected.

Therefore, in this study, we clarify the relationship between job performance and GMV measured by magnetic resonance imaging (MRI), rather than the temporary brain response measured by functional magnetic resonance imaging (fMRI) commonly adopted by brain researchers working on metacognition. GMV is thought to increase or be maintained with continued brain use (Staff et al. 2018) and has been shown to predict future executive function performance (Lee et al. 2024). To this end, from the perspective of SRL strategy theory, we examine the possibility that brain regions of interest statistically account for part of the association between job satisfaction and work performance. Job satisfaction is thought to be a function of self‐evaluation of one's job (Locke 1970) and is therefore thought to be evaluated in specific brain regions responsible for metacognition and then translated into motivation and behavior through communication with other areas. For GMV, we used the Gray Matter Brain Healthcare Quotient (GM‐BHQ) developed by Nemoto et al. (2017). Previous studies have shown that whole‐brain or regional GMV measured by the GM‐BHQ is related to psychological variables related to cognitive (Watanabe et al. 2021) and social performance (Kokubun et al. 2023). The GM‐BHQ was developed as a standardized index of GMV with a mean of 100 and a standard deviation (SD) of 15 (Nemoto et al. 2017). Previous studies have shown that GM‐BHQ declines with age and is associated with lifestyle factors and indicators of brain health. Because the present study focuses on structural brain characteristics rather than transient neural activation, GM‐BHQ was selected as an index of regional gray matter integrity. For work performance, we used absolute performance included in the health and work performance questionnaire (HPQ) developed by Kessler et al. (2003). For job satisfaction, we used items from Cammann et al. (1979).

## 2 Literature Review and Hypotheses

Metacognition is the ability to reflect on and monitor one's own cognitive processes (Dunlosky and Metcalfe 2008). Examples include planning a specific task, monitoring and understanding its progress, and evaluating and reflecting on one's performance (Dunlosky and Metcalfe 2008; Van Loon 2018). People's self‐monitoring of their learning has a significant impact on creating professional development opportunities and influences decision‐making, learning behavior, strategy use, and motivation to learn (Zimmerman 2000). Also, when self‐evaluating, people compare their performance to a certain standard, and when self‐reflecting, people make deep judgments about their learning process, motivation, beliefs, plans, and results (Van Loon 2018). However, in most work environments, opportunities for self‐monitoring, self‐assessment, and self‐reflection are limited due to the difficulty in defining personal performance standards that fit the workplace conditions (Van Loon 2018). Because people are largely unaware of their own biased self‐monitoring, they need continuous opportunities and repeated feedback to train themselves to focus on predictive cues that indicate their actual progress (Van Loon 2018). On the flip side, many workers are not given the opportunity to learn how to self‐monitor in this way and therefore do not have the skills to do so. Therefore, individual differences due to genetics and past experiences (for example, whether or not their supervisor in college taught them to train) are likely to have a significant impact on their metacognitive abilities. In light of SRL strategy theory, workers who have improved their metacognitive abilities are likely to perform better in the workplace. And in light of the idea of neuroplasticity in the brain, the brains of workers who have trained metacognitive abilities are likely to have developed brain regions that control metacognition (Cuyvers et al. 2020; Van Loon 2018).

Previous anatomical and functional neuroimaging findings have suggested the important role of the precuneus in metacognitive processing (Bader and Wiener 2024; Francis et al. 2017; Haghighi et al. 2024; McCurdy et al. 2013; Yuki et al. 2019). For example, recent studies have shown that the default mode network, including the superior frontal gyrus, precuneus, and posterior cingulate gyrus, is involved in error monitoring underlying learning and metacognition (Bader and Wiener 2024). A recent meta‐analysis revealed that both perspective‐taking and retrospective judgments on both memory and perceptual decision‐making performance depend on activation of anterior and lateral parts of the prefrontal cortex as well as activity in more caudal regions such as the premotor and precuneus (Saccenti et al. 2024). The precuneus is also involved in memory (Zou et al. 2013), reflection (Cavanna 2007), creativity (Jauk et al. 2015), and movement (Weniger et al. 2009), as well as self‐efficacy (Sugiura et al. 2016), happiness (Sato et al. 2015), and burnout (Liu et al. 2025). These are items that are addressed in the context of SRL strategy theory, because this theory is based on looking back on the past, reflecting, imagining strategies, and taking action, and it is argued that self‐efficacy supports this process (Zimmerman 2000). However, previous brain studies of metacognition have mainly focused on brain responses using fMRI, and no association between GMV in related brain regions and worker performance has been confirmed.

In addition, if the precuneus is involved in performance, other factors that determine performance must be taken into account. Although much of the literature has shown that part of workplace performance is determined by job satisfaction (Beuren et al. 2022; Gazi et al. 2024), a recent meta‐analysis has shown that the relationship between the two is moderate in effect size, and it is clear that the relationship is not completely consistent (Katebi et al. 2022). This is because emotions such as satisfaction and dissatisfaction are important incentives for behavior but are not considered to determine behavior (Locke 1970). Therefore, in light of the above‐mentioned role of the precuneus, it is possible that the precuneus may explain variance in work performance that cannot be explained by job satisfaction. In light of SRL strategy theory, it is predicted that job satisfaction is determined by one's own evaluation of one's job and that performance is determined by the generation of metacognition via the precuneus through cooperation with other brain regions that control motivation and behavior. Furthermore, because part of job satisfaction is thought to reflect the evaluation of workplace resources (Hobfoll 1989), people with high job satisfaction may be able to prevent brain resource depletion (de Andrade et al. 2016; Muraven and Baumeister 2000) and maintain the GMV of the precuneus. Therefore, in this study, we will test the following hypothesis:

Test hypothesis: Precuneus GMV statistically accounts for part of the association between job satisfaction and work performance.

In addition, considering the possibility that brain regions other than the precuneus may be related to work performance, we will conduct an exploratory analysis of the correlation between performance and the GMV of 116 brain regions measured by the GM‐BHQ to clarify the relationship between the two.

## 3 Research Methodology

### 3.1 Participants

Note that 80 participants (59 men, 21 women) aged 26 to 67 years (mean = 47.02, SD = 12.05) were recruited to the Institute of Science Tokyo from November to December 2024. The necessary number of participants was calculated using G*Power 3.1.9.7, assuming an effect size (correlation coefficient) of 0.3, equivalent to “medium” according to the Cohen (1988) criteria, an alpha error probability of 0.05, and a power (1–β error probability) of 0.8. Respondents were randomly recruited from the BHQ Consortium, a study group aiming to utilize brain information in industry. According to the self‐report, no subjects recruited had records of neurological, psychiatric, or other medical conditions that could affect the central nervous system. Brain images were taken using MRI, and the participants then answered a questionnaire. This study was approved by Ethics Committee of Institute of Science Tokyo Approval Number 2022130 and was conducted following the institute's guidelines and regulations. All methods were carried out according to the relevant guidelines, regulations, and principles of the Declaration of Helsinki. All participants provided written informed consent before participation, and their anonymity was maintained.

### 3.2 Psychological Scale

#### 3.2.1 Work Performance

The evaluation was based on responses to the Japanese version (Kawakami et al. 2020) of the item developed by Kessler et al. (2003), “Using the 0‐to‐10 scale, how would you rate your overall job performance on the days you worked during the past 4 weeks (28 days)?”

#### 3.2.2 Job Satisfaction

We used the Japanese version (Hara and Fujimoto 2020) of three items developed by Cammann et al. (1979) (e.g., All in all, I am satisfied with my job.) The Cronbach alpha reliability coefficient was 0.870.

#### 3.2.3 MRI Data Acquisition

A 3 Tesla MRI scanner (MAGNETOM Prisma, Siemens, Munich, Germany) with a 32‐channel head array coil, a three‐dimensional (3D) T1‐weighted magnetization‐prepared rapid acquisition gradient echo pulse sequence, and spin‐echo echo‐planar imaging (SE‐EPI) with generalized autocalibrated partially parallel acquisition (GRAPPA) were used for MRI data collection and structural imaging. The following parameters were used: repetition time (TR) 1900 ms, echo time (TE) 2.52 ms, inversion time (TI) 900 ms, flip angle 9°, matrix size 256 × 256, field of view (FOV) 256 mm, and slice thickness 1 mm.

#### 3.2.4 MRI Data Analysis

T1‐weighted images were segmented into gray matter (GM), white matter (WM), and cerebrospinal fluid (CSF) using Statistical Parametric Mapping 12 (SPM12; Well‐come Trust Center for Neuroimaging, London, UK) running on MATLAB R2022b (MathWorks Inc., Sherborne, MA, USA). Segmented GM images were then spatially normalized using diffeomorphic anatomical registration using the exponential lie algebra (DARTEL) algorithm (Ashburner 2007), which included incorporating a modulation step into the preprocessing model and smoothing images using a Gaussian kernel with a full width at half maximum (FWHM) of 8 mm. The smoothed GM images were then converted to proportional GM images by dividing by the intracranial volume (ICV) and used to create mean and SD images. By averaging this information and the local GM quotients using the automatic anatomical labeling (AAL) atlas, the GM‐BHQ was created with a mean of 100 and an SD of 15 points.

## 4 Analysis and Findings

All statistical analyses were performed using IBM SPSS Statistics/AMOS Version 26 (IBM Corp., Armonk, NY, USA). First, descriptive statistics and correlation analyses were conducted for the main study variables. We then tested the hypothesized indirect association among job satisfaction, precuneus GMV, and self‐rated work performance using path analysis. The mediation model was estimated using maximum likelihood estimation with 5000 bootstrap samples and 95% bias‐corrected confidence intervals. To evaluate the robustness of the findings, additional sensitivity analyses were conducted using 10,000 bootstrap samples under alternative covariate specifications (Table A1).

In addition to the hypothesis‐driven analysis focusing on the precuneus, exploratory analyses were conducted to examine whether other brain regions might also be associated with job satisfaction or self‐rated work performance. Specifically, Spearman's correlation coefficients and partial correlation coefficients were calculated between the GMVs of 116 anatomical regions and the outcome variables. Partial correlations controlled for demographic variables.

Because the Shapiro–Wilk test indicated that job satisfaction and self‐rated work performance were not normally distributed, Spearman's ρ was used throughout the exploratory analyses. For the mediation analysis, the conventional two‐sided significance threshold of *p* < 0.05 was adopted. For the exploratory analyses involving 116 regions, a more conservative threshold of *p* < 0.001 was used to reduce the likelihood of false‐positive findings arising from multiple comparisons. In addition, results with *p* < 0.01 in either hemisphere or *p* < 0.05 in both hemispheres were reported as exploratory observations that may warrant future investigation but were not interpreted as definitive evidence.

**FIGURE 1:**
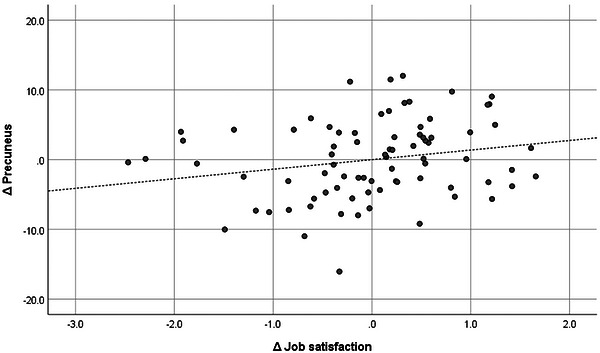
Scatter plot of job satisfaction and precuneus (*r* = 0.322). Both axes show the residuals from a regression on demographic variables (sex, age, BMI, education, managerial, professional, and technical) as independent variables. The dashed line is the regression line.

**FIGURE 2:**
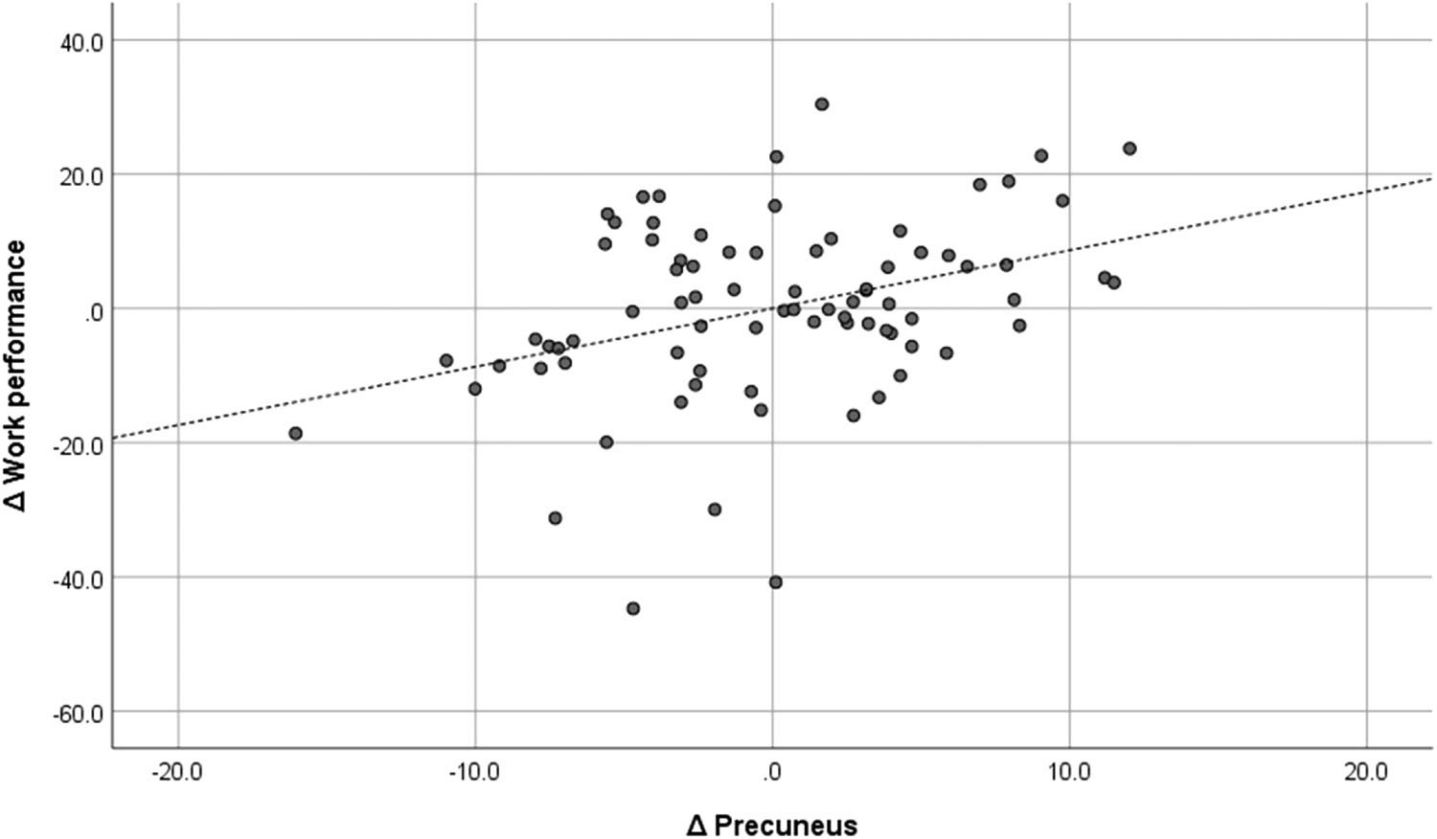
Scatter plot of precuneus and work performance. (*r* = 0.421). Both axes show the residuals from a regression on demographic variables (sex, age, BMI, education, managerial, professional, and technical) as independent variables. The dashed line is the regression line.

**FIGURE 3:**
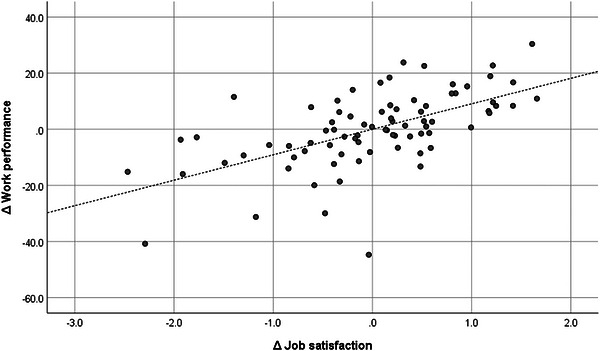
Scatter plot of job satisfaction and work performance. (*r* = 0.663). Both axes show the residuals from a regression on demographic variables (sex, age, BMI, education, managerial, professional, and technical) as independent variables. The dashed line is the regression line.

Table 1 shows descriptive statistics. Table 2 shows correlation coefficients between the subscales and total scale of GMV and psychological scales. Below the diagonal are ordinary correlation coefficients, while above are partial correlation coefficients controlling for demographic variables. The partial correlations between the three main variables are shown in Figures 1, 2, 3. Table 3 shows the results of the mediation test by path analysis. A significant direct effect was shown between job satisfaction and work performance (*r* = 0.549, *p* = 0.000). In addition, precuneus showed a significant indirect effect mediating job satisfaction and work performance (*r* = 0.054, *p* = 0.017). These results indicate that the precuneus statistically accounts for part of the association between job satisfaction and job performance, supporting the hypothesis of this study.

**TABLE 1:** Descriptive statistics.

	Mean	SD
Main variables		
Precuneus gray matter volume	98.18	8.52
Job satisfaction	3.84	0.92
Performance	7.40	1.38
Demographic variables		
Age	47.03	12.05
BMI	23.64	3.74
Years of education	17.05	2.07
	N	%
Sex		
Male	59	73.8
Female	21	26.3
Occupation		
Managerial	20	25.0
Professional/technical	37	46.3
Administrative	11	13.8
Service	7	8.8
Sales	3	3.8
Security	1	1.3
Construction/mining	1	1.3
Length of service		
Less than 1 year	2	2.3
1–3 years	5	5.7
3–5 years	14	16.1
5–10 years	16	18.4
10–20 years	13	14.9
More than 20 years	30	34.5

*Note*: BMI: body mass index computed from height and weight.

**TABLE 2:** Results of correlation analysis.

			1	2	3	4	5	6	7	8
1	Work performance	*r*		0.663***	0.421***					
		*p*		0.000	0.000					
2	Job satisfaction	*r*	0.654***		0.322**					
		*p*	0.000		0.005					
3	Precuneus	*r*	0.211	0.230*						
		*p*	0.061	0.040						
4	Sex	*r*	0.114	0.166	0.400***					
		*p*	0.315	0.141	0.000					
5	Age	*r*	0.121	0.001	−0.681***	−0.188				
		*p*	0.285	0.996	0.000	0.094				
6	BMI	*r*	0.073	−0.070	−0.474***	−0.491***	0.379**			
		*p*	0.518	0.539	0.000	0.000	0.001			
7	Education	*r*	0.002	−0.085	0.325**	−0.028	−0.336**	−0.100		
		*p*	0.988	0.454	0.003	0.802	0.002	0.377		
8	Managerial	*r*	−0.122	−0.046	−0.253*	−0.213	0.290**	0.164	−0.289**	
		*p*	0.281	0.684	0.024	0.058	0.009	0.145	0.009	
9	Professional and technical	*r*	0.125	0.123	0.026	−0.041	−0.097	0.074	0.326**	−0.536***
		*p*	0.271	0.277	0.822	0.721	0.391	0.515	0.003	0.000

*Note*: Below the diagonal are the usual correlation coefficients without controlling. Above the diagonal are the partial correlation coefficients with controlling demographic variables (sex, age, BMI, education, managerial, professional, and technical). Both are Spearman's ρ. BMI: body mass index computed from height and weight.

*n*  =  80; ^*^ *p* < 0.05; ^**^ *p* < 0.01; ^***^ *p* < 0.001.

**TABLE 3:** Results of mediation effect analysis using path analysis.

Relationship	Direct				Indirect				Conclusion
	Effect	Confidence interval		*p*‐value	Effect	Confidence interval		*p*‐value	
		Lower	Upper			Lower	Upper		
		Bound	Bound			Bound	Bound		
Job satisfaction → Precuneus → Work performance	0.549	0.351	0.709	0.000***	0.054	0.008	0.137	0.017*	Partial mediation

*Note*: All coefficients are standardized (β). Precuneus and work performance were controlled for demographic variables (sex, age, BMI, education, managerial, professional, and technical). Indirect effects were estimated as the product of the standardized path coefficients (a × b), and statistical significance was evaluated using bias‐corrected bootstrap 95% confidence intervals.

*n*  =  80; *p < 0.05; ***p < 0.001

Sensitivity analyses were conducted using 10,000 bootstrap samples under alternative covariate specifications (Table A1). The indirect association through precuneus GMV remained significant in both the full covariate model (indirect effect β = 0.054, 95% CI [0.004, 0.135], *p* = 0.028) and the age‐and‐sex‐only model (indirect effect β = 0.040, 95% CI [0.000, 0.101], *p* = 0.047). Although the indirect effect was attenuated and became non‐significant in the unadjusted model (indirect effect β = 0.014, 95% CI [−0.020, 0.067], *p* = 0.445), the overall pattern of results was broadly consistent across specifications. These findings suggest that the observed indirect association is not attributable to a specific set of covariates.

Next, we conducted an exploratory partial correlation analysis for the 116 domains, which are the smallest unit of the GM‐BHQ. The results are shown in Table A2 in the Appendix. Here again we evaluate partial correlation coefficients. Consistent with our a priori hypothesis regarding the precuneus, both Precuneus_L (*r* = 0.385, *p* = 0.0007) and Precuneus_R (*r* = 0.377, *p* = 0.0009) showed significant associations with work performance at the conservative threshold of *p* < 0.001. Four other regions, Cingulum_Mid_L (*r* = 0.231, *p* = 0.047), Cingulum_Mid_R (*r* = 0.275, *p* = 0.018), Parietal_Sup_L (*r* = 0.251, *p* = 0.031), and Parietal_Sup_R (*r* = 0.302, *p* = 0.009), met *p* < 0.05 on both the left and right, suggesting a relationship with work performance. On the other hand, no regions met the conservative criterion of *p* < 0.001 for job satisfaction. However, the regions that met the more lenient criterion of bilateral *p* < 0.05 were: Frontal_Med_Orb_L (*r* = 0.318, *p* = 0.006), Frontal_Med_Orb_R (*r* = 0.294, *p* = 0.011), Rectus_L (*r* = 0.309, *p* = 0.007), Rectus_R (*r* = 0.295, *p* = 0.011), Amygdala_L (*r* = 0.276, *p* = 0.017), Amygdala_R (*r* = 0.307, *p* = 0.008), Precuneus_L (*r* = 0.289, *p* = 0.013), Precuneus_R (*r* = 0.301, *p* = 0.009), Pallidum_L (*r* = 0.244, *p* = 0.036), Pallidum_R (*r* = 0.234, *p* = 0.045), Temporal_Sup_L (*r* = 0.277, *p* = 0.017), and Temporal_Sup_R (*r* = 0.236, *p* = 0.043). Only bilateral precuneus showed a significant correlation with both work performance and job satisfaction at a relaxed criterion of *p* < 0.05.

## 5 Discussion

The results of this study showed that the precuneus is related to job performance. Although the exploratory analysis examined 116 regions, bilateral precuneus was the only region that satisfied the a priori conservative threshold (*p* < 0.001) and was also theoretically specified based on previous literature. Formal family‐wise error corrections such as Bonferroni adjustment were not applied because the exploratory analysis was intended to generate hypotheses for future research rather than to establish definitive regional effects.

In order for a person to maintain high performance, it is necessary to repeat a series of cycles: reflecting on past successes and failures, creating, linking them to action, and reflecting. And what supports the permanence of this cycle is the learner's self‐efficacy (Zimmerman 2000). This idea is best reflected by the learning theory, SRL strategy theory, which has been confirmed to be related to high performance in work and learning (Cuyvers et al. 2020; DiBenedetto and Zimmerman 2010). Since the precuneus is related to the ability to participate in this series of processes, including metacognition (Bader and Wiener 2024; Francis et al. 2017; Haghighi et al. 2024; McCurdy et al. 2013; Yuki et al. 2019), the results of this study showing that this region is related to job performance are reasonable.

**FIGURE 4:**
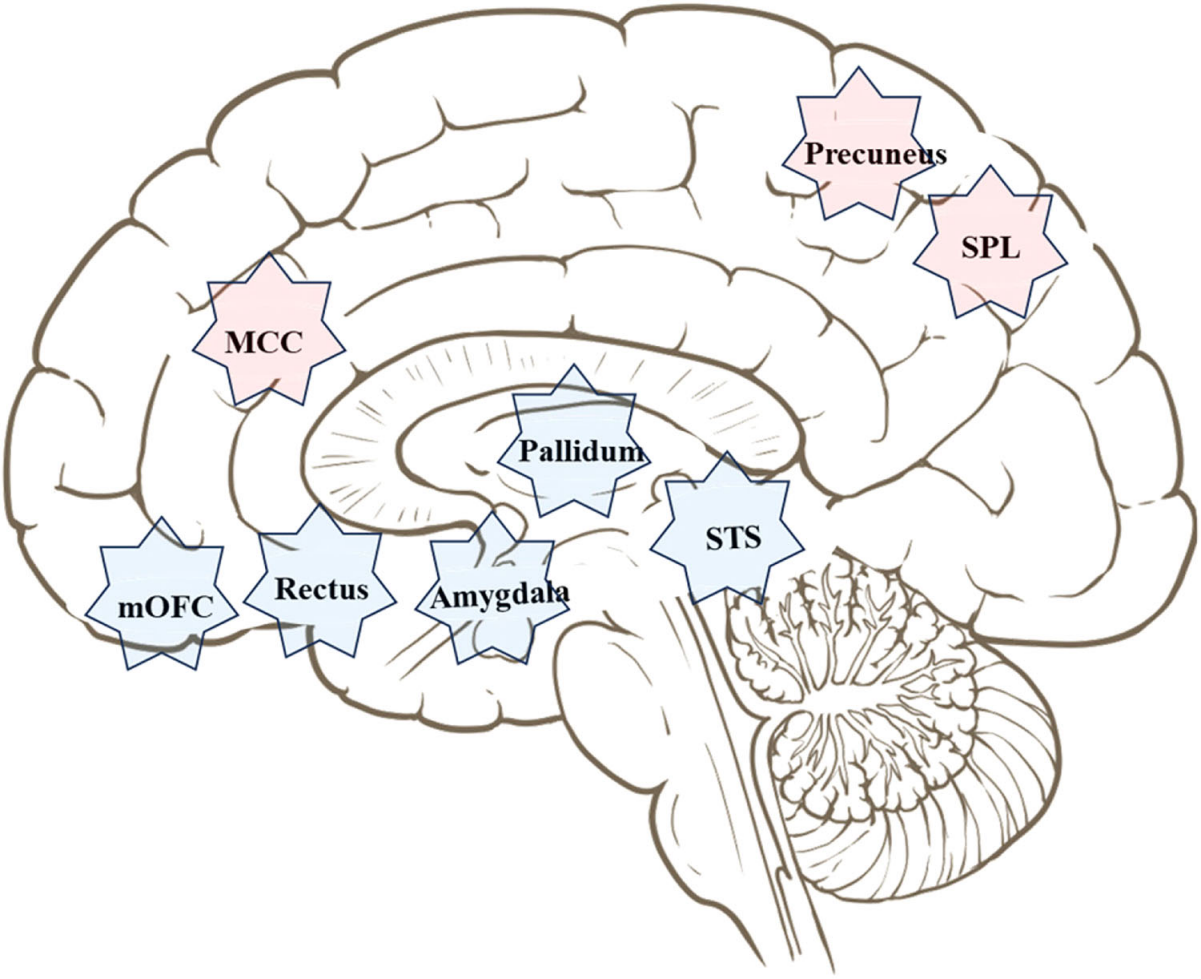
Brain regions that were found to be related to work performance or job satisfaction in this study. Red stars indicate correlation with work performance, and blue stars indicate correlation with job satisfaction. MCC, midcingulate cortex; mOFC, medial orbitofrontal cortex; SPL, superior parietal lobule; STS, superior temporal sulcus.

It was also shown that the superior parietal lobule, which is involved in memory (Crocco et al. 2018), visuospatial attention (Kumral et al. 2023), phonological decoding (Banaszkiewicz et al. 2021), and executive function (Wang et al. 2015), and the midcingulate cortex, which is involved in emotion regulation (Etkin et al. 2011), may each be related to work performance. In addition, the superior temporal sulcus, which is involved in speech language processing (Carter 2019), theory of mind (Leroy et al. 2015), face perception (Direito et al. 2019), and audiovisual integration of face and voice (Leroy et al. 2015); the rectus, which is involved in language and memory retrieval (Joo et al. 2016) and mood processing (Accolla et al. 2016); the medial orbitofrontal cortex (mOFC), which is involved in reward value monitoring, learning, and memory (Kringelbach and Rolls 2004); the amygdala, which is involved in social connections (Bickart et al. 2011); and the pallidum, which is involved in the control of voluntary movements (Gillies et al. 2017), have been shown to be related to job satisfaction. In light of the discussion of neuroplasticity, it is suggested that people who frequently use and develop these brain regions related to sensation, memory, learning, social connections, and motor control have positive self‐evaluations in the workplace, have high job satisfaction, and perform well. These regions may work together to determine job satisfaction and performance, as exemplified by recent findings that connectivity between the precuneus and mOFC is a key neural substrate of metacognitive ability (Francis et al. 2017) and occupational burnout (Liu et al. 2025). Therefore, the results of this study showing that the precuneus, which is related to metacognitive abilities, works in conjunction with these brain regions to statistically account for part of the association between both job satisfaction and work performance are reasonable. Figure 4 shows the brain regions that were found to be related to work performance or job satisfaction in this study. Sensitivity analyses suggested that the indirect association was most evident after accounting for demographic and occupational characteristics, indicating that the observed relationship is unlikely to be solely attributable to individual differences in age, sex, or occupational composition.

## 6 Limitation

This study has several limitations. First, the study was based on a cross‐sectional design, and all variables were measured at a single time point. Therefore, the present findings do not allow causal inferences regarding the relationships among job satisfaction, precuneus GMV, and self‐rated work performance. Although the statistical analyses were consistent with an indirect association through precuneus GMV, alternative explanations and reverse causal relationships remain possible. Future longitudinal and experimental studies are needed to clarify the temporal ordering of these variables.

Second, work performance was assessed using a self‐rated measure rather than an objective performance indicator. Self‐rated performance may reflect individuals’ perceptions, self‐evaluations, or response tendencies in addition to actual job performance. Therefore, caution is warranted when interpreting the findings as evidence of differences in objective work performance. Future studies should incorporate supervisor ratings, peer evaluations, or objective performance metrics.

Third, the sample consisted of individuals from a wide range of occupations. Because the meaning and criteria of high work performance may differ substantially across occupational contexts, participants may have used different standards when evaluating their own performance. Although occupational categories were statistically controlled, occupational heterogeneity may still have influenced the observed associations. Future research should examine whether similar relationships are observed within more homogeneous occupational groups.

Fourth, the sample size was relatively modest (*N* = 80), particularly for mediation analyses involving neuroimaging measures. Although bootstrap procedures supported the robustness of the observed indirect association, larger samples are needed to provide more stable parameter estimates and to improve generalizability.

Fifth, although the precuneus is known to be involved in a variety of cognitive and psychological functions, the present study did not include behavioral measures that could help explain the mechanisms underlying the observed association. For example, metacognition, self‐efficacy, memory, and related cognitive processes have been linked to precuneus function and may represent potential pathways connecting job satisfaction and work performance. Future studies should incorporate such measures to better understand the psychological mechanisms associated with precuneus GMV.

Finally, the sample used in this study was drawn exclusively from Japan, and cultural, organizational, and occupational characteristics may differ across countries. Replication in diverse cultural and organizational settings would help determine the generalizability of the present findings.

## 7 Conclusion

This study showed that the GMV of the precuneus statistically accounts for part of the association between job satisfaction and work performance. Furthermore, exploratory analysis showed that the superior parietal lobule and midcingulate cortex may be related to work performance, and the superior temporal sulcus, rectus, mOFC, amygdala, and pallidum may be related to job satisfaction. These results are the first to show that the GMV of brain regions, including the precuneus, which is responsible for functions such as metacognition, reflection, creativity, and self‐efficacy, is related to job satisfaction and work performance, consistent with SRL strategy theory.

## Author Contributions

**Keisuke Kokubun**: writing – original draft, formal analysis, software. **Yoshinori Yamakawa**: writing – review and editing, funding acquisition, supervision. **Kiyotaka Nemoto**: resources, writing – review and editing, supervision.

## Funding

This work was funded by the ImPACT Program of the Council for Science, Technology and Innovation (Cabinet Office, Government of Japan) and supported by JSPS KAKENHI (Grant Number JP17H06151; JP22K01695; JP25K15384).

## Ethics Statement

This study was approved by the Ethics Committee of Institute of Science Tokyo (Approval Number 2022130) and was conducted following the institute's guidelines and regulations. All participants provided written informed consent before participation, and their anonymity was maintained.

## Consent

All participants gave consent for the publication of the results of this study.

## Conflicts of Interest

The authors declare no conflicts of interest.

## Data Availability

The datasets generated during the current study are not publicly available but are available from the corresponding author upon reasonable request.

## 1

**TABLE A1:** Sensitivity analyses of the indirect association.

Model	Direct effect	Indirect effect	Confidence interval	*p*‐value
Full covariates	0.549	0.054	(0.004, 0.135)	0.028*
Age and sex only	0.568	0.040	(0.000, 0.101)	0.047*
No covariates	0.583	0.014	(−0.020, 0.067)	0.445

*Note*: All coefficients are standardized (β). Sensitivity analyses were conducted using 10,000 bootstrap samples. The full covariate model included age, sex, body mass index (BMI), education, and occupational categories. The age‐and‐sex‐only model included age and sex as covariates. Indirect effects were estimated as the product of the standardized path coefficients (a × b), and statistical significance was evaluated using bias‐corrected bootstrap 95% confidence intervals.

*n * =  80; **p* < 0.05.

**TABLE A2:** Exploratory correlation analysis results.

	Correlation coefficient						Partial correlation coefficient					
	Work performance			Job satisfaction			Work performance			Job satisfaction		
	*r*	*p*		*r*	*p*		*r*	*p*		*r*	*p*	
Precentral_L	0.030	0.791		0.106	0.348		0.118	0.316		0.131	0.267	
Precentral_R	−0.017	0.883		0.096	0.397		0.096	0.414		0.111	0.347	
Frontal_Sup_L	−0.132	0.245		0.108	0.341		−0.076	0.521		0.111	0.348	
Frontal_Sup_R	−0.134	0.237		0.024	0.829		−0.039	0.743		0.039	0.742	
Frontal_Sup_Orb_L	−0.010	0.928		0.167	0.139		0.074	0.532		0.198	0.091	
Frontal_Sup_Orb_R	0.008	0.944		0.130	0.252		0.110	0.353		0.160	0.173	
Frontal_Mid_L	−0.071	0.530		−0.012	0.918		0.023	0.847		−0.029	0.807	
Frontal_Mid_R	−0.032	0.775		0.071	0.529		0.049	0.677		0.078	0.511	
Frontal_Mid_Orb_L	−0.040	0.723		0.076	0.503		0.023	0.848		0.044	0.711	
Frontal_Mid_Orb_R	−0.048	0.670		0.108	0.342		0.054	0.649		0.104	0.376	
Frontal_Inf_Oper_L	0.021	0.851		0.165	0.143		0.135	0.252		0.217	0.063	
Frontal_Inf_Oper_R	0.072	0.524		0.239	0.032	*	0.155	0.187		0.259	0.026	*
Frontal_Inf_Tri_L	−0.055	0.627		0.110	0.330		0.014	0.908		0.125	0.290	
Frontal_Inf_Tri_R	−0.125	0.271		0.067	0.552		−0.069	0.560		0.064	0.590	
Frontal_Inf_Orb_L	−0.026	0.816		0.173	0.125		0.070	0.554		0.209	0.074	
Frontal_Inf_Orb_R	−0.047	0.681		0.198	0.079		0.046	0.696		0.255	0.029	*
Rolandic_Oper_L	−0.062	0.584		0.137	0.225		0.017	0.885		0.174	0.139	
Rolandic_Oper_R	−0.041	0.716		0.126	0.266		−0.005	0.968		0.122	0.301	
Supp_Motor_Area_L	0.016	0.891		0.087	0.443		0.102	0.389		0.093	0.428	
Supp_Motor_Area_R	−0.023	0.839		0.168	0.136		0.078	0.507		0.261	0.025	*
Olfactory_L	0.131	0.248		0.215	0.056		0.270	0.020	*	0.300	0.009	**
Olfactory_R	0.024	0.830		0.173	0.126		0.140	0.234		0.227	0.051	
Frontal_Sup_Medial_L	−0.026	0.816		0.158	0.161		0.079	0.502		0.199	0.089	
Frontal_Sup_Medial_R	−0.022	0.849		0.119	0.292		0.103	0.382		0.164	0.164	
Frontal_Med_Orb_L	0.073	0.523		0.239	0.032	*	0.196	0.093		0.318	0.006	**^b^
Frontal_Med_Orb_R	0.061	0.593		0.206	0.067		0.200	0.087		0.294	0.011	*^b^
Rectus_L	0.048	0.670		0.226	0.044	*	0.170	0.148		0.309	0.007	**^b^
Rectus_R	0.059	0.604		0.206	0.066		0.174	0.137		0.295	0.011	*^b^
Insula_L	−0.049	0.665		0.173	0.126		0.047	0.693		0.239	0.040	*
Insula_R	−0.115	0.310		0.108	0.338		−0.050	0.671		0.140	0.233	
Cingulum_Ant_L	0.038	0.738		0.138	0.221		0.174	0.139		0.174	0.139	
Cingulum_Ant_R	0.015	0.898		0.059	0.601		0.118	0.316		0.053	0.653	
Cingulum_Mid_L	0.079	0.486		0.179	0.112		0.231	0.047	*^b^	0.244	0.036	*
Cingulum_Mid_R	0.083	0.466		0.136	0.230		0.275	0.018	*^b^	0.194	0.097	
Cingulum_Post_L	0.056	0.625		0.144	0.202		0.176	0.133		0.178	0.129	
Cingulum_Post_R	0.093	0.414		0.116	0.305		0.191	0.104		0.114	0.332	
Hippocampus_L	0.032	0.778		0.145	0.200		0.103	0.383		0.164	0.163	
Hippocampus_R	0.051	0.652		0.169	0.135		0.127	0.281		0.185	0.115	
ParaHippocampal_L	−0.045	0.693		0.112	0.322		0.038	0.749		0.137	0.243	
ParaHippocampal_R	0.008	0.943		0.150	0.185		0.130	0.268		0.223	0.056	
Amygdala_L	0.000	0.998		0.210	0.062		0.077	0.514		0.276	0.017	*^b^
Amygdala_R	0.041	0.716		0.210	0.062		0.193	0.100		0.307	0.008	**^b^
Calcarine_L	−0.009	0.935		0.017	0.884		0.086	0.465		−0.036	0.760	
Calcarine_R	−0.010	0.933		0.018	0.875		0.060	0.611		−0.017	0.887	
Cuneus_L	−0.076	0.502		−0.064	0.572		0.004	0.975		−0.107	0.366	
Cuneus_R	−0.120	0.287		−0.072	0.525		−0.048	0.684		−0.102	0.389	
Lingual_L	−0.050	0.658		0.118	0.297		0.029	0.805		0.123	0.295	
Lingual_R	−0.007	0.954		0.044	0.695		0.095	0.423		0.034	0.771	
Occipital_Sup_L	−0.081	0.476		0.000	0.998		−0.014	0.903		−0.055	0.639	
Occipital_Sup_R	−0.158	0.160		−0.087	0.444		−0.103	0.383		−0.098	0.405	
Occipital_Mid_L	−0.001	0.994		0.088	0.438		0.122	0.299		0.101	0.391	
Occipital_Mid_R	−0.015	0.893		0.044	0.699		0.108	0.358		0.070	0.555	
Occipital_Inf_L	0.077	0.497		0.074	0.512		0.218	0.063		0.083	0.482	
Occipital_Inf_R	0.035	0.757		0.014	0.902		0.134	0.256		0.027	0.821	
Fusiform_L	0.046	0.687		0.145	0.200		0.227	0.051		0.199	0.089	
Fusiform_R	0.013	0.909		0.202	0.072		0.155	0.188		0.283	0.015	*
Postcentral_L	0.090	0.429		0.216	0.055		0.235	0.044	*	0.274	0.018	*
Postcentral_R	−0.019	0.866		0.157	0.164		0.065	0.580		0.200	0.087	
Parietal_Sup_L	0.120	0.291		0.263	0.018	*	0.251	0.031	*^b^	0.314	0.006	**
Parietal_Sup_R	0.228	0.042	*	0.182	0.107		0.302	0.009	**^b^	0.190	0.105	
Parietal_Inf_L	0.079	0.486		0.168	0.136		0.205	0.080		0.216	0.064	
Parietal_Inf_R	0.080	0.478		0.167	0.138		0.165	0.159		0.147	0.211	
SupraMarginal_L	−0.092	0.419		0.057	0.617		0.002	0.986		0.082	0.488	
SupraMarginal_R	−0.053	0.642		0.096	0.395		0.034	0.773		0.103	0.381	
Angular_L	0.026	0.817		0.087	0.444		0.111	0.348		0.072	0.543	
Angular_R	0.028	0.806		0.093	0.410		0.151	0.200		0.078	0.508	
Precuneus_L	0.219	0.051		0.237	0.035	*	0.385	0.001	***^a^	0.289	0.013	*^b^
Precuneus_R	0.188	0.094		0.211	0.060		0.377	0.001	***a	0.301	0.009	**^b^
Paracentral_Lobule_L	0.020	0.858		0.003	0.982		0.091	0.443		−0.036	0.763	
Paracentral_Lobule_R	0.073	0.521		0.202	0.073		0.160	0.173		0.252	0.030	*
Caudate_L	0.004	0.971		0.123	0.278		0.092	0.438		0.132	0.261	
Caudate_R	0.017	0.879		0.136	0.229		0.120	0.307		0.158	0.180	
Putamen_L	0.008	0.945		0.165	0.144		0.122	0.301		0.256	0.028	*
Putamen_R	−0.030	0.793		0.127	0.261		0.091	0.441		0.209	0.074	
Pallidum_L	0.106	0.350		0.175	0.122		0.209	0.073		0.244	0.036	*^b^
Pallidum_R	0.090	0.426		0.161	0.152		0.207	0.078		0.234	0.045	*^b^
Thalamus_L	−0.029	0.799		0.099	0.383		−0.011	0.923		0.067	0.572	
Thalamus_R	−0.012	0.919		0.097	0.392		−0.005	0.969		0.063	0.594	
Heschl_L	−0.110	0.331		0.136	0.229		−0.096	0.414		0.120	0.308	
Heschl_R	−0.019	0.865		0.130	0.249		0.023	0.843		0.125	0.288	
Temporal_Sup_L	−0.054	0.632		0.220	0.049	*	−0.014	0.903		0.277	0.017	*^b^
Temporal_Sup_R	0.021	0.853		0.176	0.119		0.175	0.136		0.236	0.043	*^b^
Temporal_Pole_Sup_L	0.057	0.616		0.218	0.052		0.141	0.231		0.255	0.029	*
Temporal_Pole_Sup_R	−0.075	0.511		0.110	0.333		−0.021	0.859		0.114	0.332	
Temporal_Mid_L	0.017	0.879		0.140	0.214		0.140	0.234		0.188	0.109	
Temporal_Mid_R	0.023	0.838		0.139	0.218		0.178	0.129		0.205	0.080	
Temporal_Pole_Mid_L	0.043	0.706		0.221	0.049	*	0.109	0.356		0.274	0.018	*
Temporal_Pole_Mid_R	−0.044	0.700		0.156	0.166		0.026	0.827		0.225	0.054	
Temporal_Inf_L	0.072	0.524		0.166	0.141		0.205	0.080		0.236	0.043	*
Temporal_Inf_R	−0.099	0.384		0.085	0.453		−0.018	0.879		0.131	0.264	
Cerebelum_Crus1_L	0.071	0.532		0.196	0.082		0.146	0.216		0.187	0.110	
Cerebelum_Crus1_R	−0.026	0.819		0.158	0.162		0.019	0.873		0.148	0.207	
Cerebelum_Crus2_L	−0.064	0.574		0.071	0.534		−0.075	0.525		−0.006	0.960	
Cerebelum_Crus2_R	−0.020	0.857		0.122	0.282		0.003	0.977		0.092	0.437	
Cerebelum_3_L	−0.144	0.203		−0.152	0.178		−0.133	0.259		−0.200	0.087	
Cerebelum_3_R	−0.022	0.845		0.027	0.810		−0.011	0.925		−0.003	0.983	
Cerebelum_4_5_L	−0.047	0.676		0.011	0.922		−0.021	0.857		−0.029	0.808	
Cerebelum_4_5_R	0.041	0.715		0.081	0.477		0.092	0.434		0.061	0.607	
Cerebelum_6_L	0.023	0.840		0.098	0.389		0.091	0.440		0.078	0.507	
Cerebelum_6_R	−0.015	0.892		0.094	0.407		0.048	0.684		0.083	0.482	
Cerebelum_7b_L	−0.018	0.876		0.051	0.651		0.020	0.863		−0.019	0.873	
Cerebelum_7b_R	−0.016	0.886		0.062	0.586		0.037	0.751		0.021	0.859	
Cerebelum_8_L	0.030	0.790		0.088	0.440		0.094	0.427		0.061	0.605	
Cerebelum_8_R	0.011	0.920		0.088	0.440		0.066	0.578		0.054	0.650	
Cerebelum_9_L	0.031	0.785		−0.031	0.782		0.084	0.478		−0.061	0.604	
Cerebelum_9_R	0.044	0.698		−0.020	0.858		0.122	0.300		−0.046	0.697	
Cerebelum_10_L	0.133	0.239		0.190	0.091		0.262	0.024	*	0.210	0.073	
Cerebelum_10_R	0.010	0.929		0.055	0.627		0.098	0.407		0.024	0.838	
Vermis_1_2	−0.088	0.438		−0.107	0.345		−0.052	0.660		−0.083	0.480	
Vermis_3	−0.057	0.615		−0.024	0.833		−0.043	0.716		−0.043	0.719	
Vermis_4_5	0.042	0.713		0.089	0.431		0.071	0.546		0.112	0.342	
Vermis_6	−0.023	0.842		0.074	0.512		−0.018	0.877		0.074	0.529	
Vermis_7	−0.066	0.559		0.058	0.609		−0.078	0.509		0.032	0.787	
Vermis_8	−0.074	0.513		0.086	0.446		−0.061	0.604		0.062	0.602	
Vermis_9	0.032	0.781		0.074	0.517		0.074	0.530		0.043	0.713	
Vermis_10	0.022	0.849		0.062	0.587		0.036	0.762		0.031	0.795	

*Note*: Figures are Spearman's ρ. Partial correlation coefficients were controlled for demographic variables (sex, age, BMI, education, managerial, professional and technical). BMI: body mass index computed from height and weight.

^a^Significant by conservative criteria (*p* < 0.001).

^b^Significant by relaxed criteria (*p* < 0.05 in both left and right regions).

*n*  =  80; **p* < 0.05; ***p* < 0.01; ****p* < 0.001.
